# Impact of Frailty and Sarcopenia on Thirty-Day and Long-Term Mortality in Patients Undergoing Elective Endovascular Aortic Aneurysm Repair: A Systematic Review and Meta-Analysis

**DOI:** 10.3390/jcm13071935

**Published:** 2024-03-27

**Authors:** François Saucy, Hervé Probst, Johan Hungerbühler, Coralie Maufroy, Jean-Baptiste Ricco

**Affiliations:** 1 Service de Chirurgie Vasculaire, Ensemble Hospitalier de la Côte, Hôpital de Morges, 1110 Morges, Switzerland; herve.probst@ehc.vd.ch (H.P.); johan.hungerbuhler@ehc.vd.ch (J.H.); coralie.maufroy@ehc.vd.ch (C.M.); 2 Ecole de Médecine, Université de Poitiers, 86073 Poitiers, France; jean.baptiste.ricco@univ-poitiers.fr

**Keywords:** EVAR, sarcopenia, frailty

## Abstract

**Background:** The aim of this study was to assess the prognostic role of frailty and sarcopenia on the survival of patients with AAA undergoing elective endovascular repair (EVAR). **Methods:** A systematic review of the literature was conducted in accordance with Meta-analysis of Observational Studies in Epidemiology (MOOSE). The association of frailty or sarcopenia with 30-day mortality and late survival was expressed as odds ratios (ORs) or hazard ratios (HRs) with a 95% confidence interval (CI). Meta-analysis random effects models were applied. The five-factor modified frailty index (mFI-5) was used as a frailty metric and sarcopenia was determined using computed tomography angiography (CTA) with measurements of the total psoas muscle area. Frailty was defined as patients with mFI-5 ≥ 0.6 and sarcopenia was defined as the total psoas muscle area (TPA) within the lowest tertile. **Results:** Thirteen observational cohorts reporting a total of 56,756 patient records were eligible for analysis. Patients with frailty (mFI-5 ≥ 0.6) had significantly increased 30-day mortality than those without frailty (random effects method: OR, 4.84, 95% CI 3.34–7.00, *p* < 0.001). Patients with sarcopenia (lowest TPA tertile) had significantly increased 30-day mortality according to the fixed effects method (OR, 3.30, 95% CI 2.17–5.02, *p* < 0.001), but not the random effects method (OR, 2.64, 95% CI 0.83–8.39, *p* = 0.098). Patients with sarcopenia or frailty had a significantly increased hazard ratio (HR) for late mortality than those without frailty or sarcopenia according to the random effects method (HR, 2.39, 95% CI 1.66–3.43, *p* < 0.001). The heterogeneity of the studies was low (I^2^: 0.00%, *p* = 0.86). The relation of frailty to age extracted from four studies demonstrates that the risk of frailty increases with age according to the random effects method (standard mean differences, SMD, 0.52, 95% CI 0.44–0.61, *p* < 0.001). The heterogeneity of the studies was low (I^2^: 0.00%, *p* = 0.64). **Conclusions:** Patients with sarcopenia or frailty have a significantly increased risk of mortality following elective EVAR. Prospective studies validating the use of frailty and sarcopenia for risk prediction after EVAR are needed before these tools can be used to support decision making.

## 1. Introduction

Endovascular aneurysm repair (EVAR) has become the preferred method of AAA repair [1]. Compared with open AAA repair, EVAR is less invasive with lower short-term mortality rates [2,3,4]. As such, EVAR has particular utility in patients with suitable anatomy who are at risk for postoperative adverse outcomes, particularly those who are considered ineligible for open surgical repair (OSR), such as elderly patients with impaired functional status [5].

Impaired functional status with dependence for activities of daily living is a component of frailty and has been associated with worse postoperative outcomes in previous studies [6,7]. Dependent functional status and frailty, including the five-factor modified frailty index and sarcopenia, are associated with increased mortality after EVAR and OSR [8]. However, the actual effect of functional status on outcomes after EVAR has been identified as a research priority [9].

Impaired functional status with dependence for activities of daily living is a defining component of frailty. The five-factor modified frailty index (mFI-5) used in this study has been shown to predict mortality after open aortic surgery. However, postoperative mortality is low after EVAR [1], and the first objective of this study was to determine whether mFI-5 could predict 30-day and late mortality after elective EVAR.

In parallel, the second objective of the study was to examine the correlation of sarcopenia measured as total psoas muscle area (TPA) with 30-day mortality and long-term survival in patients undergoing EVAR.

Numerous tools have been used to assess frailty in surgical patients. Additionally, sarcopenia can be assessed using cross-sectional imaging and has been used as a proxy measure for frailty [10]. EVAR patients are frequently elderly with multiple comorbidities, and frailty may be highly prevalent among them. According to EUROSTAT, for the first half of the 21st century, the proportion of the population over 60 years will increase from 20% in 2000 to 33% in 2050. Although EVAR provides less trauma, some frail elderly patients will still suffer from major postoperative complications, poor quality of life, and death. Frailty and sarcopenia are crucial components of geriatric assessment and cause vulnerability to adverse outcomes [11]. The aim of the present systematic review and meta-analysis was to evaluate the quality of evidence of frailty and sarcopenia on the short- and long-term outcomes following elective EVAR.

## 2. Methods

The protocol of the study was prepared in accordance with the Meta-analysis of Observational Studies in Epidemiology (MOOSE) checklist [12]. The Population, Intervention, Comparator, and Outcomes (PICO) framework was used to define the research question (Table 1).

### 2.1. Seach Strategies

The Medline, Embase, Cochrane, and Scopus databases were searched from January 2010 to December 2023 for articles investigating frailty in patients undergoing elective endovascular aortic aneurysm repair (EVAR). The search results were combined using EndNote 21 and duplicate references were removed.

### 2.2. Study Selection

**Figure 1:**
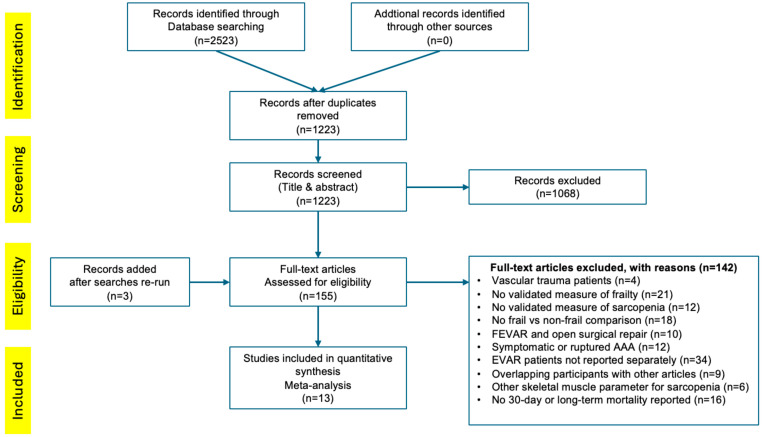
PRISMA flowchart of the literature search to identify studies on the effect of frailty or sarcopenia on mortality in patients undergoing elective endovascular aortic aneurysm repair (EVAR).

Two authors (J.-B.R. and F.S.) independently screened titles and abstracts (Figure 1). From 1223 articles, 155 full texts were screened by two other authors (H.P. and J.H.). Studies that included a defined and validated measure of preoperative frailty and sarcopenia in patients undergoing elective EVAR with 30-day mortality as the endpoint with the number of events in each group (frail and non-frail) were included in the analysis. Studies reporting long-term follow-up in patients undergoing elective EVAR with hazard ratio (HR) and a validated measure of frailty were also included in the analysis.

Studies reporting odds ratios (ORs) without the number of patients at risk in each group (frail and non-frail) were excluded, as were the studies that included vascular trauma and studies whose data for either frail or non-frail patients were not reported separately.

A descriptive narrative of the results was undertaken for all studies included in the systematic review (Table 2). The studies’ designs and dates of inclusion were compared to ensure that the results for the same patients were not included more than once in the meta-analysis.

### 2.3. Frailty Assessment Tools

Currently, multiple methods are proposed for assessing frailty as a predictive tool for postoperative outcomes. In this study, we only selected studies using validated methods.

### 2.4. Five-Factor Modified Frailty Index (mFI-5)

The mFI-5 used in this study has been validated for use as a frailty metric across surgical specialties [26] and uses the following five criteria: (1) diabetes mellitus; (2) congestive heart failure; (3) hypertension (HTN) requiring antihypertensive medication; (4) chronic obstructive pulmonary disease; (5) functional level of dependence in performing activities of daily living prior to surgery such as bathing, dressing, feeding, and using the toilet. Patients were considered functionally impaired if they were partially or totally dependent for their daily activities. The mFI-5 score for each patient was determined by dividing the number of criteria present by the total number of variables assessed (n/5). As previously reported, a score of 0.6 or greater on the mFI-5 was indicative of frailty [16]. Studies using other methods for measuring frailty, such as the Risk Analysis Index (RAI) [14] and other measures of preoperative functional status, were converted to mFI-5 scores.

### 2.5. Sarcopenia

Sarcopenia was determined radiologically using computed tomography angiography (CTA) with measurements of the TPA by summing the cross-sectional area of the left and right psoas muscles at the level of third to fifth lumbar vertebrae (L3–L5) [21,24,27]. The TPAs was divided into three even tertiles, with the lowest tertile used as the definition of sarcopenia [24].

### 2.6. Short-Term and Long-Term Outcomes

Short-term outcomes with 30-day mortality and frailty were reported in eight cohorts, including four with mFI-5 criteria and four with sarcopenia. Long-term outcomes with survival (minimum of 2 years) were reported in four studies, one with mFI-5 criteria and three with sarcopenia (TPA within the lowest tertile). In addition, the association of frailty or sarcopenia with patient age was further investigated in four studies.

### 2.7. Meta-Analysis

Data from 16 studies that reported the patient factors of 30-day or late mortality following elective EVAR for frail and non-frail patients separately were included in the meta-analysis. Studies that did not define a cut-off value for frailty (mFI-5 criteria or sarcopenia) were excluded from the meta-analysis.

For short-term (30-day) outcomes, generally expressed as dichotomous data, the number of events in frail and non-frail groups were entered as odds ratios (ORs) with a 95% confidence interval (CI) using the Mantel–Haenszel method [28]. Long-term outcomes were entered as HRs with a 95% confidence interval (CI) using the generic inverse variance method [29].

Random effect models were used to pool data due to the expected heterogeneity of the included studies and were compared with the fixed effect model. The inverse variance method was used to combine the adjusted odds ratios (ORs) and assess the association with 30-day mortality. The effect estimates were reported with 95% confidence intervals (CIs) and presented as forest plots. The chi-square heterogeneity test was used and expressed as I^2^ statistics for overall heterogeneity. A funnel plot was presented to assess bias in the publications, with the frailty effect on the horizontal axis and standard error on the vertical axis [30]. The vertical line representing the estimated OR was derived using a fixed effect meta-analysis, and the two diagonal lines represent 95% confidence limits (effect ± 1.96 SE) around the summary effect for each standard error (SE) on the vertical axis. In the absence of heterogeneity, 95% of the studies lie within the funnel defined by these diagonal lines. Medcalc Statistical Software v. 22.016 (Medcalc Sotfware Ltd., Ostend, Belgium) was used to perform the statistical calculations.

## 3. Results

In this systematic review and meta-analysis, 56,756 patient records were retrieved from the systematic literature search in 13 articles identified as meeting the selection criteria.

### 3.1. Frailty and 30-Day Mortality

**Figure 2:**
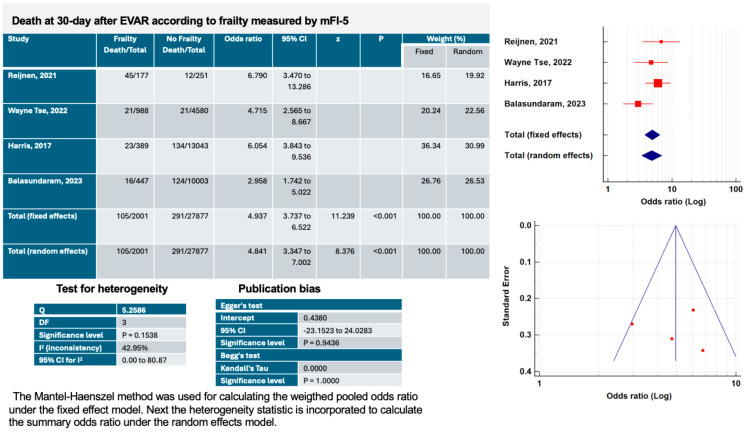
Main characteristics of the four studies included in the systematic review and meta-analysis on effects of frailty (mFI-5 ≥ 0.6) on 30-day mortality after elective endovascular aortic aneurysm repair (EVAR).

Frailty (mFI-5 ≥ 0.6) was associated with a significantly increased 30-day mortality in patients who underwent elective EVAR (Figure 2) according to the fixed effects method (OR, 4.93, 95% CI 3.73–6.52, *p* < 0.001) and the random effects method (OR, 4.84, 95% CI 3.34–7.00, *p* < 0.001) [13,14,15,16]. The heterogeneity of the studies was moderate (I^2^: 42.9%, *p* = 0.15). The risk of publication bias was low according to Egger’s test (intercept 0.43, *p* = 0.94) and Begg’s test (Kendall’s Tau: 0.0000, *p* = 1.00).

In addition to 30-day mortality, frailty was also found to predict hospital stay > 30 days (*p* = 0.02), readmission (*p* < 0.001), and prolonged intensive care unit stay > 3 days (*p* < 0.001). In one study [16], the readmission rate went from 5% in non-frail patients to 12.3% in patients with mFI-5 (0.6–1.0). Harris et al. [15] found 4% of systemic complications in non-frail patients vs. 13% in frail patients (*p* < 0.001), and Tse et al. [14] found 8% of systemic complications in non-frail patients vs. 13% in frail patients (*p* < 0.001).

In two studies, return to the operating theatre (OT) was also analyzed. Tse et al. [14] found return to OT in 3% of non-frail patients and in 7% of frail patients (*p* = 0.001). Harris et al. [15] found return to OT in 4% of non-frail patients and in 7% of frail patients (*p* = 0.001).

### 3.2. Sarcopenia and 30-Day Mortality

**Figure 3:**
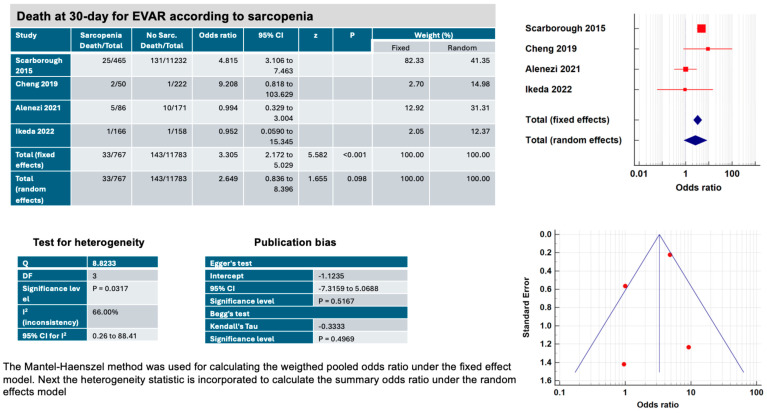
Main characteristics of the four studies included in the systematic review and meta-analysis on effects of sarcopenia (total psoas muscle area within the lowest tertile), on 30-day mortality after elective endovascular aortic aneurysm repair (EVAR).

Sarcopenia, defined as TPA within the lowest tertile, was associated with a significantly increased 30-day mortality in patients who underwent elective EVAR (Figure 3) according to the fixed effects method (OR, 3.30, 95% CI 2.17–5.02, *p* < 0.001), but not the random effects method (OR, 2.64, 95% CI 0.83–8.39, *p* = 0.098) [17,18,19,20]. The heterogeneity of the studies (I^2^: 66.0%, *p* = 0.03) is partially related to the different methods of measurement of the psoas cross-sectional area at different levels (L3–L5) and may explain the difference observed between fixed and random effects. The risk of publication bias remained acceptable according to Egger’s test (intercept 0.51, *p* = 0.51) and Begg’s test (Kendall’s Tau: −0.3333, *p* = 0.49).

### 3.3. Long-Term Effect on Mortality of Frailty and Sarcopenia

**Figure 4:**
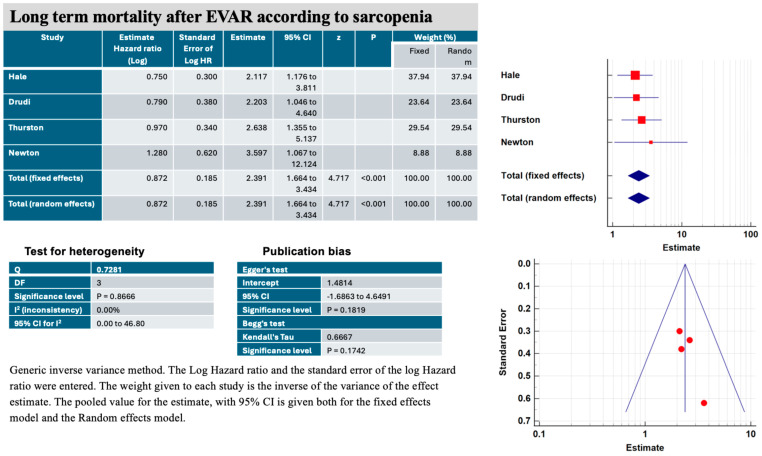
Main characteristics of the four studies included in the systematic review and meta-analysis on effects of sarcopenia (total psoas muscle area within the lowest tertile), on long-term mortality after elective endovascular aortic aneurysm repair (EVAR).

Sarcopenia (TPA within the lowest tertile) and frailty (mFI-5 ≥ 0.6) were associated with a significantly increased hazard ratio (HR) for late mortality in patients who underwent elective EVAR (Figure 4) [21,22,23,24]. This risk was doubled with the fixed effects method (estimated HR, 2.39, 95% CI 1.66–3.43, *p* < 0.001) and with the random effects method (estimated HR, 2.39, 95% CI 1.66–3.43, *p* < 0.001). The heterogeneity of the studies was low (I^2^: 0.00%, *p* = 0.86). The risk of publication bias was low according to Egger’s test (intercept 1.48, *p* = 0.18) and Begg’s test (Kendall’s Tau: 0.6667, *p* = 0.17).

### 3.4. Association of Age with Frailty or Sarcopenia

**Figure 5:**
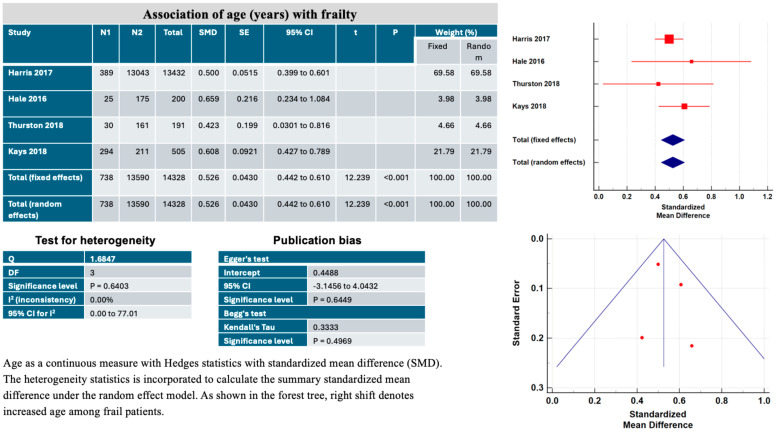
Main characteristics of the four studies included in the systematic review and meta-analysis showing the association of age (years) with frailty (mFI-5 ≥ 0.6) or sarcopenia (total psoas muscle area within the lowest tertile) in patients undergoing elective endovascular aortic aneurysm repair (EVAR).

The relation of frailty to age (Figure 5) was extracted from four studies limited to EVAR [15,21,23,25]. The results are presented as standardized mean difference (SMD) with 95% CI for age. If the value 0 is not within the 95% CI, then the SMD is statistically significant. Cohen’s rule for the interpretation of the SMD statistics is that a value of 0.2 indicates a small effect, and a value above 0.5 indicates a large effect. The meta-analysis demonstrates that the risk of frailty or sarcopenia increases with age with the fixed effects method (SMD, 0.52, 95% CI 0.44–0.61, *p* < 0.001) and the random effects method (SMD, 0.52, 95% CI 0.44–0.61, *p* < 0.001). The heterogeneity of the studies was low (I^2^: 0.00%, *p* = 0.64). As shown in the funnel plot, the risk of publication bias was low according to Egger’s test (intercept 0.44, *p* = 0.64) and Begg’s test (Kendall’s Tau: 0.3333, *p* = 0.49).

## 4. Discussion

This study examined whether frailty and sarcopenia affect treatment outcomes after elective EVAR in patients with AAA. Frailty and sarcopenia were found to affect postoperative and late survival.

This meta-analysis was limited to studies including EVAR patients because, to date, no meta-analysis has specifically studied the impact of frailty and sarcopenia on EVAR patients. Numerous studies have already shown the prognostic value of frailty and sarcopenia on the survival of patients with open AAA repair, or in series, including open repair of AAA and EVAR but without specifying the influence of frailty and sarcopenia in each of these groups analyzed separately [8,22,31,32,33,34].

Despite being minimally invasive and associated with lower morbidity and mortality compared to open AAA repair, this meta-analysis demonstrates that EVAR remains a procedure with an increased risk of mortality for patients with impaired functional status, particularly in the setting of advanced age. Harris et al. [15] showed that dependent patients aged 80 years or older with chronic renal or pulmonary disease presented with a substantially increased risk. This subset of patients should be considered during preoperative evaluation and could benefit from targeted readaptation to improve outcomes.

Analyses of NSQIP data indicate that frailty is an independent risk factor for death after a range of surgical procedures [6,7,35], despite advances that have further reduced the invasiveness of surgery.

These findings are consistent with recent studies considering physical fitness and frailty among patients undergoing open or endovascular aortic repair [8,31]. Wang et al. [33], in a systematic review including 22 cohort studies, found that overall frailty assessed as functional status was associated with a significantly increased 30-day mortality risk (OR 5.1) after open and endovascular aortic repair. In the same meta-analysis, sarcopenia predicted long-term mortality (HR 2.1). Antoniou et al. [36], in another meta-analysis including seven observational cohorts, found a significant link between sarcopenia and death after AAA repair (HR 1.7), but with only two studies including patients who received EVAR. Interestingly, in a study of nursing home patients undergoing open AAA repair or EVAR, Beffa et al. [37] found that preoperative frailty scores were associated with subsequent recovery, whereas repair modality was not.

Functional status measured by preoperative mFI-5 or sarcopenia is a useful marker of perioperative risk. It is easily assessed and could potentially help to improve the functional status of the patient and physiological reserve with structured exercises [27,38,39]. However, further studies are required to determine whether such interventions are beneficial for EVAR patients.

In addition, functional dependence, as one mFI-5 criterion, is associated with complications independently of major comorbidities. Dependence could lead to complications through non-ambulatory condition and poor nutrition. Although not assessed in this meta-analysis, impaired nutritional status and cognitive function could also increase the risk of complications among dependent patients.

## 5. Limitations

This meta-analysis has several limitations. Frailty and sarcopenia were assessed in observational studies with intraobserver and interobserver variability in the estimation of these factors. Furthermore, the lack of standardized evaluation methods for sarcopenia based on CT imaging increases bias in the measurement of outcomes, and potential confounder stratification, including age, gender, height, and body mass index were not always reported. However, objective preoperative measurements such as CTA psoas muscle size for sarcopenia, even carried out retrospectively, is arguably not subject to a high risk of bias. In addition, the ease of assessing functional status with mFI-5 and its constant relationship with outcomes in other datasets suggest that it remains a valuable measure even if retrospectively assessed.

Another limitation of this meta-analysis is the lack of granularity of many datasets that do not report details of all risk factors, including the diameter of the aortic aneurysm. In the setting of frailty and sarcopenia, our results suggest that prophylactic repair starting at 5.5 cm AAA [1] warrants further prospective studies. Along those lines, data must be interpreted with caution, as long-term survival is affected by many factors in patients undergoing EVAR.

## 6. Conclusions

This meta-analysis demonstrated that elective EVAR remains a procedure of risk in older patients with sarcopenia or impaired functional status measured by mFI-5 criteria, both of which are easily assessed preoperatively. However, until now, we have not determined whether this assessment of frailty is useful, as no controlled studies have shown that structured exercise and rehabilitation in frail patients could improve survival after EVAR.

## Figures and Tables

**Table 1:** Population, Intervention, Comparator and 0utcomes (PICO) framework.

Framework	Meaning
Population (P)	Patients with any component of frailty spectrum or sarcopenia
Intervention (I)	Endovascular aneurysm repair (EVAR)
Comparator (C)	Patients who are not frail
Outcomes (0)	Mortality at 30-day and long-term survival
Research question	What is the impact of frailty or sarcopenia on survival?
Studies evaluated	Studies reporting on outcomes (mortality) after EVAR in patients who have associated frailty or sarcopenia and in patients without frailty or sarcopenia

**Table 2:** Characteristics of studies included in final analysis.

** Main Author/Year **	** Study Type **	** Procedure **	** Frailty or Sarcopenia** **with/without **	** Endpoint **
Reijnen 2021 [13]	Retrospective Single centre	EVAR	Frailty-mFI-5* 177/251	Death 30-day
Tse 2022 [14]	Retrospective Single centre	EVAR	Frailty-mFI-5* 988/4580	Death 30-day
Harris 2017 [15]	Retrospective NSQIP 2010–2014	EVAR	Frailty-mFI-5* 389/13043	Death 30-day
Balasundaram 2023 [16]	Retrospective NSQIP 2015–2019	EVAR	Frailty-mFI-5* 447/10003	Death 30-day
Scarborough 2015 [17]	Retrospective NSQIP 2005–2010	EVAR	Sarcopenia* 465/11232	Death 30-day
Cheng 2019 [18]	Retrospective Single centre	EVAR	Sarcopenia* 50/222	Death 30-day
Alenezi 2021 [19]	Retrospective Single centre	EVAR	Sarcopenia* 86/171	Death 30-day
Ikeda 2022 [20]	Retrospective Single centre	EVAR	Sarcopenia* 166/158	Death 30-day
Hale 2016 [21]	Retrospective Single centre	EVAR	Sarcopenia* 25/175	Death > 2 years
Drudi 2016 [22]	Retrospective Single centre	EVAR	Sarcopenia* 49/100	Death > 2 years
Thurston 2018 [23]	Retrospective Multi-centre	EVAR	Sarcopenia* 30/161	Death > 2 years
Newton 2018 [24]	Retrospective Single centre	EVAR	Sarcopenia* 45/89	Death > 2 years
Kays 2018 [25]	Retrospective Single centre	EVAR	Sarcopenia* 294/211	Death > 2 years

mFI-5*: Patients with frailty defined as patients with mFI-5 ≥ 0.6 or dependent with assistance for activities of daily living including feeding, toileting, bathing, dressing and mobility. Sarcopenia*: Patients with sarcopenia defined as lowest tertile for total psoas muscle area adding the right and left psoas muscle area. Number of patients with sarcopenia/number of patients without sarcopenia.
